# *Staphylococcus lugdunensis* Pacemaker-related Infection

**DOI:** 10.3201/eid1108.041177

**Published:** 2005-08

**Authors:** Harald Seifert, Dirk Oltmanns, Karsten Becker, Hilmar Wisplinghoff, Christof von Eiff

**Affiliations:** * University of Cologne, Cologne, Germany;; † University of Münster Hospital and Clinics, Münster, Germany

**Keywords:** Staphylococcus lugdunensis, Small colony variants, Pacemakerrelated infection, Colony variation, Device-related infection, Recurrent infection, Persistence

## Abstract

We report the first known case of a device-related bloodstream infection involving *Staphylococcus lugdunensis* small-colony variants. Recurrent pacemaker-related bloodstream infection within a period of 10 months illustrates the poor clinical and microbiologic response even to prolonged antimicrobial drug therapy in a patient infected with this staphylococcal subpopulation.

During the past decade, *Staphylococcus lugdunensis* has emerged as an important pathogen implicated in both community-acquired and nosocomial infections (1,2). Clinical manifestations of infections with these organisms include abscesses (3), meningitis (4), ventriculoperitoneal shunt infection (5), spondylodiscitis (6), prosthetic joint infection (7), catheter-related bacteremia (2), and endocarditis (1). Infections with *S. lugdunensis* tend to have a more fulminant course, with an outcome resembling that of *S. aureus* infections rather than that caused by coagulase-negative staphylococci (8). In addition, these organisms are frequently misidentified as *S. aureus* because of their morphologic appearance with yellow pigmentation and complete hemolysis when cultured on blood agar.

Small-colony variants (SCVs) are mainly reported in *S. aureus*, and interest in infections with SCVs has recently increased after an association between recovery of *S. aureus* SCVs and persistent and relapsing infection has become evident (9). SCVs are a slow-growing subpopulation of the species with characteristics that can associated by a common factor, i.e., alterations in electron transport (10). The generation time for SCVs is up to 9-fold longer than for metabolically normal strains, which results in tiny colonies that are frequently not visible until after 48 to 72 hours of incubation. Consequently, correct identification and susceptibility testing for clinical laboratories are complicated, which may result in diagnostic underestimation and therapeutic failures. While most studies have dealt with SCVs of *S. aureus*, little is known about infections with SCVs of coagulase-negative staphylococci. Recently, 2 cases of bloodstream infections caused by SCVs of *S. epidermidis* and *S. capitis*, respectively, were reported (11). Both infections were related to foreign bodies and observed after pacemaker implantation.

We report the first known case of a device-related bloodstream infection due to *S. lugdunensis* SCVs and other colony variants of this species. Of particular interest, this infection was also observed after pacemaker implantation.

## The Case

In July 2003, a 61-year-old man was transferred from a local hospital to our cardiothoracic surgery department with a diagnosis of pacemaker lead infection. Past medical history included nephrectomy in 1996 for cancer of the left kidney and implantation of a universal demand pacemaker (dual chamber pacemaker) for treatment of sick sinus syndrome in 1990. In August 2002, after being in place for 12 years, the pacemaker battery was replaced. Three months later, the patient was admitted to a local hospital with a temperature of 40°C and chills. Laboratory findings included a leukocyte count of 17,500/μL and a C-reactive protein (CRP) level of 90 mg/L. A transesophageal echocardiogram showed thickening of the left coronary aortic valve, and thrombotic material was seen on the ventricular pacemaker lead. A blood culture drawn on admission showed *S. lugdunensis* susceptible by agar diffusion to penicillin, oxacillin, erythromycin, clindamycin, rifampin, and aminoglycosides. Antimicrobial drug therapy was instituted with intravenous ampicillin/sulbactam and gentamicin for 14 days with prompt resolution of clinical symptoms, and follow-up blood cultures remained negative. Three days later, however, a spiking fever and chills developed in the patient. Antimicrobial drug treatment was changed to intravenous vancomycin and rifampin. The patient's condition improved rapidly, and he was discharged after 3 weeks of antimicrobial drug therapy when the CRP value had returned to normal.

**Figure 1:**
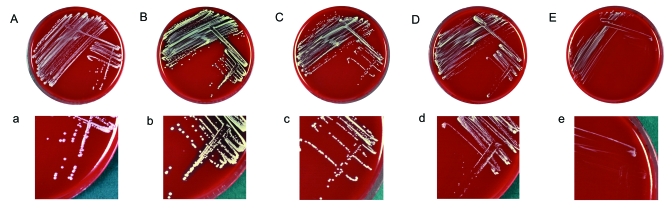
Sheep blood agar plates (A–E) and magnified sectors (a–e) after overnight incubation at 37°C showing different morphotypes of clonal isolates of the Staphylococcus lugdunensis strain recovered from blood cultures and the infected pocket of a patient with pacemaker infection. Plates A–D/a–d show S. lugdunensis colonies exhibiting the normal phenotype characterized by colonies of different diameter, ranging from 0.8 to 2.5 mm with creamy (A/a) or yellow (B–D/b–d) pigmentation and moderately heavy (B/b), weak (C–D/c–d), or absent (A/a) hemolysis; plate E/e shows the small-colony variant phenotype characterized by tiny (pinpoint), nonpigmented, and nonhemolytic colonies.

Two months later in February 2003, the patient was readmitted to the cardiology department with the presumptive diagnosis of endocarditis. During a transient febrile episode, a blood culture was obtained that again yielded *S. lugdunensis* (Figure 1A). Antimicrobial drug therapy was resumed with intravenous flucloxacillin and gentamicin. All 4 follow-up blood cultures obtained 3 and 4 days later, when the patient was afebrile, were again positive for *S. lugdunensis*. An echocardiogram did not show vegetations or other evidence of endocarditis. Pacemaker removal was strongly suggested, but the patient refused. After 14 days of intravenous treatment, the antimicrobial drug regimen was changed to oral administration of flucloxacillin for 14 days. After a full recovery, the patient was discharged, but removal of the pacemaker system was recommended if clinical symptoms reappeared.

Four months later in July 2003, the patient came to the local hospital with recurrent high fever and chills, a leukocyte count of 12,200/μL, and a CRP value of 37 mg/L, but he did not show any peripheral sign of endocarditis. Four sets of blood cultures drawn on admission showed *S. lugdunensis*. A transesophageal echocardiogram showed large vegetations in the right atrium inserting at the ventricular lead but no involvement of cardiac valves. The patient responded promptly to the initiation of antimicrobial drug therapy with intravenous flucloxacillin and gentamicin and became afebrile. He was then transferred to our cardiothoracic surgery department for pacemaker ablation.

Four days later, the complete pacemaker system, including the intracardiac leads, was removed by open heart surgery. The cardiac valves did not show signs of infective endocarditis, but large vegetations adhered to both the atrial and the ventricular lead. Follow-up blood cultures remained negative but thrombotic material scraped from the pacemaker leads was analyzed by culture. After 2 days of incubation, this material yielded nonhemolytic and nonpigmented, as well as yellow-pigmented, hemolytic colonies of variable size, which were gram-positive catalase-positive cocci, consistent with staphylococci. The results of subcultures on solid media suggested a mixed population of staphylococci, with at least 4 different colony morphologies (Figure 1B–E). Four single-colony subcultures of different colony morphotypes also produced colony variations that persisted in serial subcultures of single colonies.

Clumping factor was not present and tube coagulase test results were negative. Identification was initially attempted with the gram-positive identification card provided with the VITEK 2 system (bioMérieux, Marcy l'Etoile, France). The large hemolytic morphotype (Figure 1B) showed a profile consistent with *S. lugdunensis*, with positive results for ornithine decarboxylase, trehalose, and l-pyrrolidonyl-β-naphthylamide. Other morphotypes were repeatedly identified as *S. haemolyticus* (Figure 1C; T index 0.93) and *S. auricularis* (Figure 1D and E; T index 0.46), respectively. The *S. lugdunensis* isolate that grew as tiny (pinpoint), nonpigmented, and nonhemolytic colonies was shown to be a hemin-auxotrophic SCV (Figure 1E). The *S. lugdunensis* isolate (large colony morphotype) was susceptible to all antimicrobial agents in the VITEK GPS-P526 card test (bioMérieux) and did not produce β-lactamase. The other morphotypes did not grow sufficiently to allow antimicrobial susceptibility testing with the VITEK system. However, susceptibility to penicillin and oxacillin was confirmed by an Etest (AB Biodisk, Solna, Sweden) for all colony variants.

**Figure 2:**
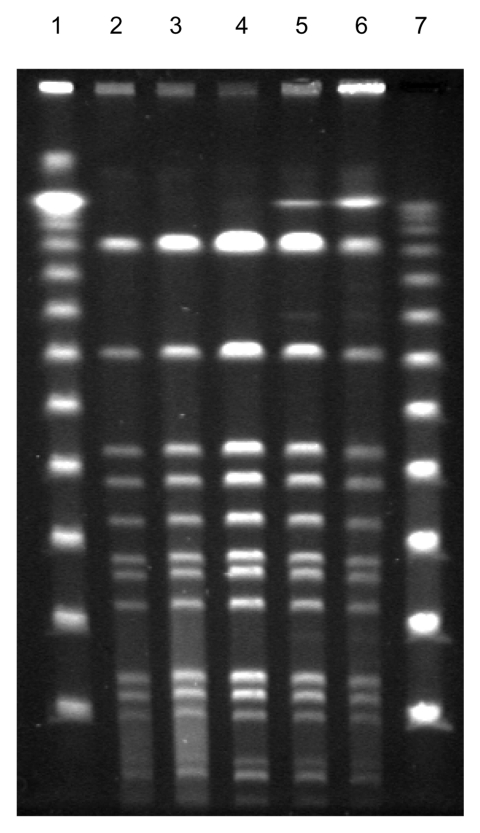
Fingerprint patterns for different Staphylococcus lugdunensis colony morphotypes, including small-colony variants (SCVs), after pulsed-field gel electrophoresis after digestion with SmaI, showing identical isolates. Lanes 1 and 7, 100-bp ladder; lane 2, blood isolate; lanes 3–5, colony variants; lane 6, SCVs of S. lugdunensis obtained from thrombotic material.

The API ID 32 Staph system (bioMérieux) identified all morphotypes as *S. lugdunensis*, which was later confirmed by 16S ribosomal RNA gene sequencing using the RIDOM entries (12). All isolates, including an additional *S. lugdunensis* blood isolate obtained in February 2003 that produced flat, white, and nonhemolytic colonies (Figure 1A), were compared by pulsed-field gel electrophoresis and found to be identical, although the colony morphology was different (Figure 2).

Postoperative recovery was uneventful. Treatment with intravenous flucloxacillin and gentamicin was continued for 14 days. A 72-hour electrocardiogram did not show any need for pacemaker reinsertion. Fourteen days after surgery, the patient was discharged from the hospital, after a total clinical course of 10 months with recurrent infections.

## Conclusions

Previous reports have rarely emphasized colony variation as an important feature of *S. lugdunensis*. In the initial description of the species in 1988 (13), colony variation was observed in 3 of 11 strains. More recently, Leung et al. reported colony variation of *S. lugdunensis* in a fatal case of endocarditis (14). Unlike other staphylococcal species such as *S. capitis* and *S. hominis*, which show colony variation that disappeared after extended incubation, mixed morphotypes of *S. lugdunensis* were persistently detectable through incubation and subculture (14). The authors speculated that preceding antimicrobial drug therapy may play a role in producing colony variation in *S. lugdunensis* and that previous studies may have underreported the characteristic of colony variation seen in this species.

Some of the aberrant morphotypes described in earlier studies may have in fact been SCVs. Both prior exposure to antimicrobial drugs and the presence of chronic or recurring infections, often with indwelling foreign devices that have been associated with SCVs of *S. aureus*, *S. epidermidis*, and *S. capitis* (1,15), are features commonly observed in infections with *S. lugdunensis* (2,4,5,7,14). In our case, repeated courses of gentamicin therapy may have selected for SCVs. Although the infection showed a rather benign clinical course and did not confirm other reports of *S. lugdunensis* endocarditis in which the infection was more aggressive, it illustrates the chronic, recurrent, and persistent nature of infections with SCVs and the problems associated with delayed identification of *S. lugdunensis* colony variants and interpretation of its clinical significance.

The refusal of the patient to have the pacemaker removed added to the chronic course of the infection. Although these variants were not identified until removal of the device, the clinical importance of SCVs for this persistent infection can be anticipated. Clinical isolates are often a mixed population of parent strains and SCVs. Because of their different generation times, even a small percentage of normally growing organisms may rapidly replace SCVs in liquid medium such as a blood culture during overnight incubation. Thus, SCVs may have gone undetected in previously obtained blood cultures. Increased awareness of colony variation and the possible occurrence of SCVs as a characteristic feature of *S. lugdunensis* should be helpful in earlier recognition of the pathogen and appropriate management of the infection.

Dr. Seifert is professor of clinical microbiology at the Institute for Medical Microbiology, Immunology and Hygiene at the University of Cologne, Germany. His research interests include the molecular epidemiology of nosocomial pathogens, in particular, *Acinetobacter* and *Staphylococcus* species, catheter-related infections, and antimicrobial drug resistance and its mechanisms.
